# Adding a twist to the loops: the role of DNA superhelicity in the organization of chromosomes by SMC protein complexes

**DOI:** 10.1042/BST20240650

**Published:** 2024-12-10

**Authors:** Antonio Valdés, Christian H. Haering

**Affiliations:** Chair of Biochemistry and Cell Biology, Biocenter, Julius-Maximilians-Universität of Würzburg, Wurzburg, Germany

**Keywords:** cohesin, condensin, DNA loop extrusion, DNA topology, SMC complex, topoisomerase

## Abstract

Structural maintenance of chromosomes (SMC) protein complexes, including cohesin, condensin, and the Smc5/6 complex, are integral to various processes in chromosome biology. Despite their distinct roles, these complexes share two key properties: the ability to extrude DNA into large loop structures and the capacity to alter the superhelicity of the DNA double helix. In this review, we explore the influence of eukaryotic SMC complexes on DNA topology, debate its potential physiological function, and discuss new structural insights that may explain how these complexes mediate changes in DNA topology.

## A short primer to DNA topology

**Figure 1.**
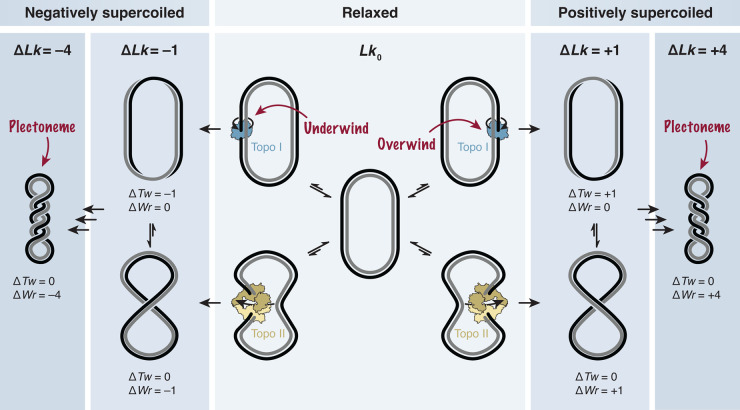
A brief overview of DNA topology. Changes in *Lk* of a closed double-stranded DNA circle (center) require cleavage of one or both DNA strands by type-I (top) or type-II (bottom) topoisomerase, respectively. Under- or overwinding of the helix by rotation or DNA double strand cross-inversion results in negatively (left) or positively (right) supercoiled DNA circles. The resulting changes in geometry are reflected by alterations of the DNA helicity (twist, top), by the formation of DNA crossovers (writhe, bottom), or a combination of the two. Multiple DNA crossovers usually converge into plectonemes.

While the double-helical structure of DNA offers remarkable stability, it also imposes a challenge: any local changes in its geometry, such as the unzipping of the strands during replication or transcription, inevitably induce long-range torsional stress. Torsional stress in the DNA double helix is most tangible for covalently closed double-stranded DNA circles, where the two strands wind around each other a set number of times, defined as the linking number (*Lk*). Altering *Lk* in these circular DNA molecules requires the breakage of one or both strands, followed by either rotation around the helical axis or passage of the DNA through the break, respectively, before the strands are rejoined. This process is managed by enzymes called topoisomerases [1] (Figure 1).

Eukaryotic cells employ two types of topoisomerases: type-1B and type-2. Type-1B topoisomerases break the phospho-deoxyribose backbone of one strand in the DNA duplex, forming a covalent bond between an active-site tyrosine and the 3′-phosphate end and leaving a free 5′-hydroxyl group on the opposite side of the break. This allows the broken strand to rotate around the intact strand, relieving torsional strain in the DNA helix without requiring additional energy [2]. In contrast, type-2 topoisomerases require the energy of adenosine triphosphate (ATP) hydrolysis to pass one DNA duplex through a temporary double-strand break in another DNA duplex. To this end, they cleave both strands of a ‘gated’ DNA segment, forming covalent bonds between their active-site tyrosine residues and the two 5′-phosphate DNA ends. This allows a ‘transported’ DNA segment to pass through the cleaved gated segment [3].

In the absence of torsional stress, the ground state linking number *Lk*_0_ equals roughly the DNA length divided by the ∼10.5 base pairs (bp) of a complete helical turn of B-form DNA [4,5]. Due to thermal fluctuations and the activity of topoisomerases, *Lk* values measured for DNA molecules isolated from cells usually follow a Gaussian distribution centered on *Lk*_0_. Overwinding of the right-handed DNA helix results in positive supercoiling (Δ*Lk* > 0), whereas underwinding results in negative supercoiling (Δ*Lk* < 0).

Whereas *Lk* can be used as a numerical description of DNA topology, it does not depict its spatial configuration. The latter can instead be described by two geometric quantities: twist (*Tw*) and writhe (*Wr*). Twist represents the number of times each DNA strand turns around the central axis of the duplex. Writhe represents the number of times the DNA axis crosses over itself to produce left-handed (*Wr* > 0) or right-handed (*Wr* < 0) gyres, giving rise to toroids or, more commonly, plectonemes (Figure 1). The topological quantity *Lk* and the geometrical quantities *Tw* and *Wr* are linked in the Călugăreanu-White-Fuller theorem [6,7]

1 Lk=Tw+Wr

Changes in *Lk* will therefore alter the spatial geometry of the DNA with respect to *Tw*, *Wr*, or both. Changes in *Wr* are usually more frequent than changes in *Tw*, since the energy requirement for the formation of plectonemes is lower than for the deformation of the DNA double helix along its central axis [8].

DNA regions under helical tension can be stabilized (‘constrained’) by the binding of proteins, or they can be freely accessible (‘unconstrained’) and hence unprotected from the action of topoisomerases. A prime example of unconstrained supercoils are those generated by transcribing RNA polymerases, which generate overwound DNA ahead of them and underwound DNA in their wake, or by the replicative helicase, which generates positive supercoils ahead of the replication fork. Whereas most of these supercoils are relaxed by type-1 or type-2 topoisomerases, some are instead diffused by replication fork rotation to its wake, resulting in the generation of double-stranded sister chromatids intertwines (SCIs, also referred to as catenanes) that can only be resolved by type-2 topoisomerases [9]. An example of constrained helical tension is the wrapping of the DNA duplex around histone octamers in 1.7 left-handed superhelical turns to create a nucleosome. Protein binding stabilizes DNA deformations (*Tw* and *Wr*) without changes in *Lk*, in contrast with the action of topoisomerases at unconstrained DNA regions. The constrained supercoiling energy can be released when the constraining factor is removed from the DNA and can impact the surrounding DNA geometry before topoisomerases have a chance to act.

## SMC protein complexes and DNA loop extrusion

**Figure 2.**
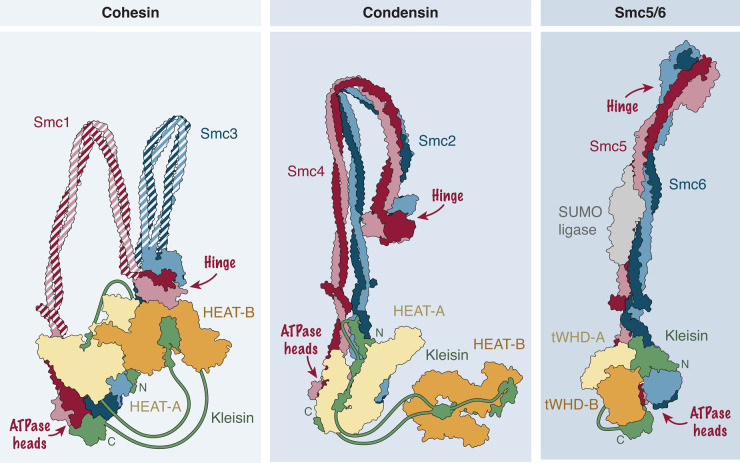
Subunit organization of the three eukaryotic SMC protein complex families. The ATPase ‘head’ domains of a heterodimer of SMC coiled-coil subunits (red and blue) are connected by the binding of the amino- (N) and carboxy-terminal (C) domains of the flexible kleisin subunit (green). In the case of cohesin and condensin, the kleisin subunit tethers two HEAT-repeat subunits (yellow and orange) to the complex. In the case of the Smc5/6 complex, the (considerably shorter) kleisin binds two tandem winged-helix subunits (yellow and orange). Structure models are built on the co-ordinates of human cohesin (PDB 6WG3), budding yeast condensin (PDB 6YVU and 5OQQ), and the six-subunit budding yeast Smc5/6 complex (PDB 8I13). Shaded coils were manually placed to connect cohesin hinge and head domains.

SMC protein complexes are essential for the large-scale organization and dynamics of chromosomes across all domains of life. Three SMC complexes exist in eukaryotes: condensin folds chromatin fibers into rod-shaped chromatids during mitosis, cohesin links sister chromatids and organizes chromosome domains during interphase, and the Smc5/6 complex functions during DNA replication, transcription, and DNA damage repair, among other processes [10]. Several variants exist for some of these complexes in different species (e.g. condensin I and II in many metazoans). Despite their distinct roles, SMC complexes share a common structural framework based on a heterodimer of ∼50-nm long coiled-coil SMC subunits connected via their ‘hinge’ domains at one end of the coils and exhibiting a pair of ATPase ‘heads’ at the other end (Figure 2). A long, flexible ‘kleisin’ subunit connects the heads even when they are not engaged by sandwiching two ATP molecules in-between their split active sites. The kleisin recruits to the complex two subunits that are composed of alpha-helical HEAT-repeat motifs in the case of cohesin and condensin or of tandem winged-helix motifs in the case of the Smc5/6 complex. Their common architecture suggests that all three complexes share the same core mechanism of action [11].

Recent studies converged on the idea that this common mechanism might be the extrusion of DNA loops [12]. Single-molecule microscopy recordings showed the successive formation of individual or sometimes convoluted DNA loops *in vitro* in the presence of purified condensin, cohesin, or Smc5/6 complexes [13–16]. The speed of loop extrusion (up to 1–2 kbp/s) correlated negatively with the tension present in the DNA substrate, and loops stalled as soon as this tension reached a force of only a few pico Newton. Correlation to measurements of ATPase rates in bulk assays suggested that SMC complexes can move several hundreds of bp during each round of their ATPase reaction cycle, fundamentally different to the 1–2 bp steps taken by other known DNA motors [11].

The detailed molecular mechanism of SMC-mediated DNA loop extrusion remains to be elucidated. Several models have been proposed to explain the loop extrusion properties observed *in vitro*, inferred from *in vivo* chromosome contact maps, or derived from *in silico* simulations [17]. Despite their differences, all models agree that DNA movements depend on (a) multiple DNA binding sites within monomers or dimers of SMC complexes, and (b) large conformational changes in the long coiled-coil SMC subunits. Yet, formal proof that DNA loop extrusion is the mechanism used by SMC complexes to shape genomes is still missing, and alternative ideas have been brought forward [18,19]. Some of these ideas are based on the ability of SMC complexes to change DNA superhelicity.

In this short review, we focus our discussion on the role of DNA supercoiling by the three eukaryotic SMC complexes condensin, cohesin, and the Smc5/6 complex. For a discussion of prokaryotic SMC complexes, we refer the reader to more comprehensive recent reviews [12,20,21].

## Do SMC complexes reshape chromosomes by introducing DNA supercoils?

One of the first observations that eukaryotic SMC complexes affect DNA topology came from the discovery that circular DNA, after incubation with condensin I affinity-purified from mitotic *Xenopus* egg extracts, retained positive supercoils or DNA crossovers after protein denaturation when the reaction mix contained type-1 or type-2 topoisomerase, respectively [22,23]. The supercoiling activity required ATP and was reduced when purified condensin had been treated with phosphatase or when cyclin-dependent kinase had been depleted from the extract [24]. A similar supercoiling activity by budding yeast condensin increased upon phosphorylation by mitotic kinases [25], as did supercoiling by condensin I purified from human cultured cells [26].

**Figure 3.**
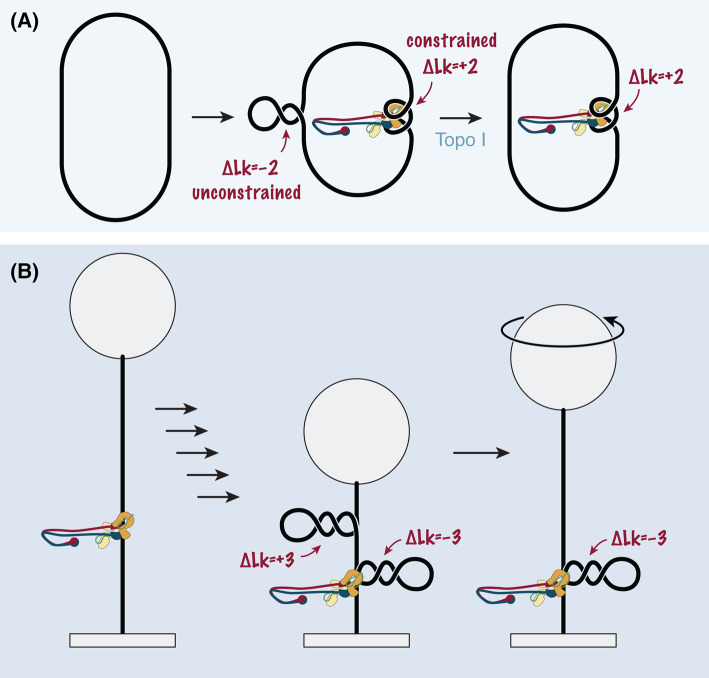
Methods to measure changes in DNA topology mediated by SMC complexes. (**A**) Bulk assays with closed DNA circles. Any changes in topology introduced locally by the SMC complex will be compensated by topology changes of the opposite sign in the non-bound DNA region. Since the latter are unconstrained, they will be removed by topoisomerases, whereas the topology changes constrained by the SMC complex will be maintained and can be detected by gel electrophoresis after protein denaturation. (**B**) In single-molecule magnetic tweezers assays, a double-stranded DNA is tethered between a surface and a paramagnetic bead. Topology changes induced by an SMC protein complex will result in compensatory changes in the non-bound DNA region. The formation of plectonemes results in the shortening of the tethered DNA. Rotation of the beads under- or overwinds the DNA in the unbound region, while DNA e.g. extruded as a loop is topologically insulated.

Assuming that the supercoils generated by budding yeast and human condensin were also positive in orientation (see below), the results of these *in vitro* experiments could be explained if condensin constrained positive toroids or plectonemes (Δ*Wr* > 0) in a reaction that is ATP-dependent and regulated by phosphorylation. While the constrained positive supercoiling would be protected from topoisomerases, the compensating unconstrained negative supercoiling in the non-bound DNA regions would be susceptible to topoisomerase cleavage (Figure 3A). Consistent with this proposal are electron spectroscopic images of small (3 kbp) circular plasmid DNAs bound by a single *Xenopus* condensin I, which showed an average of two crossovers in the non-bound DNA region and ∼120–190 bp DNA that co-localized with the protein in a shape that resembled two gyres of ∼12 nm diameter [27]. Yet, it is unclear from now available high-resolution condensin crystal [28,29] and cryo-electron-microscopy [30–32] structures how condensin could wrap DNA around one or several of its subunits as tightly as a histone octamer does. Nevertheless, condensin co-structures with DNA revealed a notable degree of bending of the double helix at the two identified DNA binding sites (see below).

Whether the formation of unconstrained negative supercoils as a consequence of wrapping DNA around condensin could account for the compaction of mitotic chromosomes [22] is questionable. Quantitative imaging of fluorescently labeled condensin on mitotic chromosomes in live human cells suggested that one condensin I or II binds every ∼80 or ∼300 kbp, respectively [33]. The density of negative supercoils that could be introduced by condensin in such a manner would presumably be far too small to explain the large-scale changes in chromosome architecture observed during mitosis. Furthermore, the ubiquitous activity of topoisomerases would most likely instantly relax any unconstrained supercoils. Finally, if the global architecture of chromosomes were determined by DNA supercoiling, the supercoiling waves generated by RNA or DNA polymerases [34] would be expected to have a much greater impact than they actually have.

Condensin-dependent changes in DNA superhelicity might, however, be important for the efficient decatenation of sister chromatids. In budding yeast, circular mini chromosomes were found to increase in positive supercoiling during mitosis after depletion of type-2 topoisomerase, and this change in topology was prevented by condensin inactivation [35]. Since positively supercoiled mini chromosomes were more efficiently decatenated than their negatively supercoiled counterparts, positive supercoiling by condensin might isolate SCIs for their efficient resolution by type-2 topoisomerases.

## Do SMC complexes act as DNA supercoiling sensors?

Different observations suggest that there exists a link between DNA superhelicity and the chromosomal localization of SMC complexes. A preference for binding supercoiled DNA might, for example, explain the enrichment of condensin I at transcription start sites in chicken [36] and human [37] cultured cells; although most transcription would probably have stalled by the time condensin I associated with chromosomes in these cells. In contrast, many genes continue to be actively transcribed during mitosis in fission yeast, and these genes are similarly enriched for condensin binding [37].

A clue for a possible role for SMC-dependent recognition of regions of supercoiling comes from the identification of one of the two type-2 topoisomerases expressed in human cells (TOP2B) as a potential cohesin interaction partner in biotin proximity labeling (BioID) experiments [38]. Recruitment of TOP2B by cohesin and the zinc-finger DNA-binding protein CTCF to chromosome domain boundaries might be necessary for the resolution of DNA intertwines that arise during cohesin-mediated DNA loop extrusion [39] (see below). This notion is supported by the finding that TOP2B inhibition increased the formation of DNA double-strand breaks at CTCF sites [40]. Notably, CTCF binding sites often demark the boundaries of ‘supercoiling domains’, which were defined as regions of ∼100 kbp median size that differ in the probability of psoralen intercalation as a measure of underwound (Δ*Tw* < 0) DNA duplexes [41].

Evidence that the Smc5/6 complex localizes to regions of positive supercoiling comes from chromatin immunoprecipitation experiments in budding yeast, which showed that Smc5/6 was frequently enriched between convergently transcribed genes (i.e. two genes that face each other) [42]; similar to what had previously been reported for cohesin [43,44]. Smc5/6 enrichment scaled with transcriptional activity and increased upon topoisomerase inactivation, presumably due to a further accumulation of positive supercoils ahead of the transcribing polymerases. This conclusion is supported by the findings that Smc5/6 dissociated from extrachromosomal DNA substrates after transcriptional repression in cultured human cells and that dissociation was reduced by simultaneous knock-down of type-1 and type-2 topoisomerases [45]. Smc5/6 is presumably likewise recruited to regions of increased supercoiling generated by DNA replication, since the density of Smc5/6 binding sites on budding yeast chromosomes scales with chromosome length, as does replication-induced helical stress [46].

Although Smc5/6 complexes affinity-purified from budding yeast cells showed no clear preference for binding supercoiled plasmid DNA of either handedness at low salt conditions [47], salt-resistant binding of recombinantly purified budding yeast Smc5/6 complexes to positively supercoiled or catenated (kinetoplast) DNA circles was more efficient than binding to negatively supercoiled or relaxed circles [48]. The same complexes preferentially bound to (and started to extrude loops at; see below) the tips of positive (Δ*Wr* > 0) but not negative (Δ*Wr* < 0) plectonemes in single-molecule microscopy experiments [42]. A similar preference for binding positive plectonemes was reported for budding yeast condensin [49].

## Do SMC complexes alter DNA superhelicity during DNA loop extrusion?

Since the extrusion of large DNA loops seems to be the activity that unites all three groups of eukaryotic SMC complexes [13,14,16], it is tempting to speculate that the formation of DNA supercoils might be intrinsically coupled to DNA loop extrusion.

**Figure 4.**
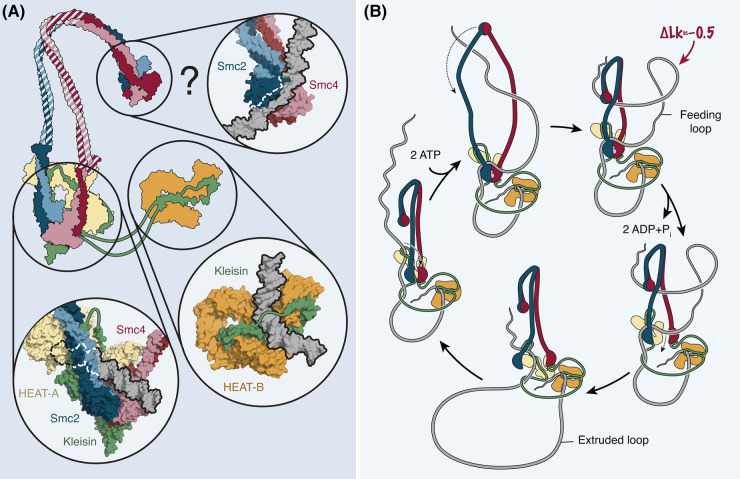
DNA binding sites in SMC complexes and their potential role in DNA loop extrusion. (**A**) Structure models of DNA bound to the engaged condensin SMC heads and the HEAT-A and kleisin subunits (PDB 7QEN) and to the HEAT-B and kleisin subunit (PDB 7QFW). AlphaFold 3 [66] generated model of the yeast Smc2–Smc4 hinge dimer bound to a 5-nt single-stranded DNA bubble. Shaded coiled coils were manually placed to indicate the connectivity of the individual structures. (**B**) DNA topology changes integrated into a ‘reel-and-seal’-type model for DNA loop extrusion [17]. ATP binding results in the formation of a feeding loop that temporarily stores Δ*Lk* ≈ −0.5 in a ‘feeding’ DNA loop delimited by binding by the SMC hinge and head domains, before merger of this loop with the ‘extruded’ DNA loop. How the feeding loop is protected from cleavage by type-2 topoisomerases is not known. For simplicity, a possible ATP-binding-induced movement of HEAT-A with the SMC hinge domain as reported for cohesin [60] is not shown.

At protein concentrations typically used for single-molecule DNA loop extrusion experiments (a few nM) [13], approximately two orders of magnitude lower than those used for the plasmid supercoiling assays discussed above, condensin was found to restrain negative instead of positive supercoils on DNA circles [50]. Since supercoiling was resistant to nuclease P1, which nicks single-stranded DNA, condensin either created a left-handed DNA turn (Δ*Wr* < 0) or, if it created untwisted DNA stretches (Δ*Tw* < 0), these would have to be constrained (protected) by the protein complex. Supercoiling furthermore depended on the presence of ATP but, surprisingly, not on the presence of the HEAT-B^Ycg1^ subunit, which rules out the possibility that the strong bend in the DNA duplex observed in a HEAT-B^Ycg1^-DNA co-structure [31,32] (Figure 4A, bottom right) might be responsible for restraining a left-handed DNA turn. Instead, a change in DNA superhelicity is more likely mediated by the DNA binding site created by the HEAT-A^Ycs4^ subunit together with the ATP-engaged SMC heads (Figure 4A, bottom left), since addition of the non-hydrolysable nucleotide analog AMP-PNP further increased the supercoiling that had initially been generated in the presence of ATP. Whereas the compensatory positive supercoils in the non-bound DNA region of the DNA circles could be resolved by type-1 topoisomerase, they were resistant to type-2 topoisomerase. Condensin must hence induce supercoiling in a manner that prevents the formation of DNA crossovers, which would serve as a type-2 topoisomerase substrate.

The results of these experiments can be integrated into a ‘pinch and merge’ model for DNA loop extrusion [50], which postulates that every condensin reaction cycle creates a small, left-handed DNA loop that is delimited by two DNA binding sites. The supercoiling of this ‘feeding loop’ increases upon ATP-dependent SMC head dimerization. Release of DNA from one of the two binding sites then allows the feeding loop to merge with an ‘extruded loop’, which was delimited by the site that had just been released and a third DNA binding site, presumably at the SMC hinge (see below).

A concentration-dependent shift from negative to positive supercoiling was also reported for similar experiments with human cohesin (preprint: [51]). Supercoiling of DNA circles required the presence of the HEAT-A^Nipbl/Scc2^ subunit but, like for condensin, not the HEAT-B^SA1^ subunit. Supercoiling was abolished by mutation of positively charged residues in the SMC hinge domain, consistent with the idea that the hinge serves as a third DNA binding site. Computational simulations provided a possible explanation for the concentration-dependent shift in the handedness of supercoiling: if only a few loop-extruding complexes bound to a plasmid, the likelihood of type-1 topoisomerases relaxing compensatory (positive) supercoils outside the (negatively supercoiled) extruded loop would be higher, whereas the opposite would be true for large numbers of loop-extruding complexes.

Negative supercoiling by SMC complexes was not only observed in bulk experiments with DNA circles, but also in single-molecule magnetic tweezers experiments (preprints: [51,52]). Addition of human cohesin, yeast condensin, or yeast Smc5/6 to a linear DNA molecule attached with one end to a surface and with the other end to a paramagnetic bead resulted in a step-wise reduction of the distance between the bead and the surface in the presence of ATP; presumably due to the extrusion of a DNA loop [53]. Changes in rotation curves before and after protein addition then allowed the determination of changes in *Lk* of the DNA outside of the protein-bound DNA domain, since the latter is topologically insulated (Figure 3B). For all three DNA loop extruders, an increase in *Lk*, even after only a single extrusion step in the presence of AMP-PNP, suggested that SMC complexes constrained a corresponding negative change in *Lk* (Δ*Lk* ≈ −0.6). Whether this change in topology was generated in the extruded DNA loop or in a ‘feeding loop’ that would also be topologically insulated from the DNA outside the protein-bound domain, remained unknown. This value matches estimates for negative supercoiling restrained by condensin in the bulk experiments with DNA circles (Δ*Lk* [−0.8, −0.4]), although these numbers depend on the assumption that all condensin molecules in the reaction bound DNA [50].

One drawback of the magnetic tweezers experiments is that protein and DNA remained invisible (preprint: [52]), which raises the question whether the observed changes in topology stem from the extrusion of a DNA loop by a single complex, or whether they might result from the loading of multiple complexes in a reaction that is stimulated by ATP hydrolysis [32,54,55]. If the former were the case, it is unclear why loops were formed at a surprisingly slow rate (<200 bp/min) [53] in the tweezers setup when compared with previous DNA loop extrusion rates measured at similar forces for relaxed (∼1 kbp/s) [13] or supercoiled DNA (∼0.4 kbp/s) [49] in single-molecule microscopy assays. It is also puzzling why mutation of the DNA binding site on the engaged cohesin SMC heads still induced efficient stepwise DNA shortening in the tweezers experiment, but almost abolished DNA loop extrusion in single-molecule microscopy assays (preprint: [51]). Finally, it remains unknown why SMC complexes locked by AMP-PNP in an SMC head-engaged state before they encountered DNA were still able to support a single-step change in DNA topology in the magnetic tweezers assay if that step depended on a conformational change induced by nucleotide binding, and why this differed from what was seen in bulk experiments with DNA circles when AMP-PNP was the only nucleotide available [50].

## How might SMC complexes induce DNA superhelicity changes during loop extrusion?

Although the identity of the DNA binding sites remained undefined in the ‘pinch and merge’ model, the main aspects of this model can be reconciled with ‘reel-and-seal’-type models [17] if the extruded loop were held between the HEAT-A/SMC heads and HEAT-B binding sites and the feeding loop were held between the HEAT-A/SMC heads binding site and a third binding site formed by the SMC hinge.

Isolated hinge dimers of different SMC complexes were found to bind single- or double-stranded DNA, with a preference for the former [56–59]. It is tempting to speculate that the negative supercoiling in a presumptive feeding DNA loop between HEAT-A/SMC heads and SMC hinge might, at least in part, result in DNA unwinding to create a single-stranded bubble (5–6 bp for Δ*Tw* ≈ −0.5) that is protected from nuclease P1 cleavage by binding the hinge (Figure 4A, top). Movement of the SMC hinge to the head domains after nucleotide binding to the SMC heads, described as a ‘swing’ motion in the case of cohesin [60], might serve to increase the superhelicity of the feeding loop (Figure 4B). If AMP-PNP locked condensin in the resulting folded state, with the hinge close to the engaged ATPase heads as seen in cohesin cryo-EM structures [61–63], it could explain why AMP-PNP trapped a state of increased supercoiling, even if the swing movement *per se* is not driven by nucleotide binding [60].

Another conformational change in SMC complexes that might contribute to DNA supercoiling becomes apparent from the comparison of nucleotide-free [30] and nucleotide- and DNA-bound [31,32] structures of the condensin SMC head domains in complex with the HEAT-A^Ycs4^ and kleisin subunits: ATP binding induces a ∼90° swivel motion of the HEAT-A^Ycs4^ amino terminus and a ∼30° rotation of the HEAT-A^Ycs4^ carboxy terminus, which, together with the formation of a DNA binding surface on the engaged SMC heads, presumably account for DNA rotation and bending at this site (Figure 4A, bottom left).

There now exists a consensus from different studies that the force-generating step for DNA loop extrusion is the ATP-mediated engagement of the two SMC head domains ([32], preprint: [52], [64]). If this step generated a small DNA ‘feeding’ loop between engaged SMC heads and hinge domains, DNA bending and rotation at the HEAT-A/SMC head interface [65] and/or a swing motion of the hinge towards the heads could increase the negative superhelicity of the DNA in this loop. The elastic energy stored in this loop could then be used for the next step in the DNA loop extrusion cycle. For ‘reel-and-seal’-type models, this next step is the disengagement of the binding site at the SMC heads upon ATP hydrolysis and/or ADP and phosphate release, and the transfer of one of the two strands of the feeding loop through the disengaged SMC heads (Figure 4B). The stored elastic energy could hereby be converted into kinetic energy to push DNA into the extruded loop.

## Perspectives

- The discovery of DNA loop extrusion by SMC protein complexes has revolutionized the concept of chromosome folding at the genomic scale. The underlying molecular mechanism is, however, still incompletely understood.
- Although the ability of SMC complexes to alter DNA superhelicity had been recognized soon after their discovery, the physiological relevance of this activity remained unclear. Recent studies suggest that DNA supercoiling might be an integral part of the DNA loop extrusion process.
- The development of new technologies like, for example, cryo-electron tomography approaches, might be able to reveal the superhelicity of DNA in loop-extruding SMC complexes. These and additional studies will need to resolve whether DNA supercoiling is merely a byproduct of the movement of SMC complexes along DNA, or whether it plays an active role in expanding DNA loops, thereby transforming DNA from a passive substrate into an active participant of the process.

## Abbreviations

ADP: adenosine diphosphate
AMP-PNP: adenosine-5′-(β,γ-imido)triphosphate
ATP: adenosine triphosphate
bp: base pair
CTCF: CCCTC-binding factor
DNA: deoxyribonucleic acid
HEAT: Huntingtin, elongation factor 3, protein phosphatase 2A, TOR1
kbp: kilo base pair
nt: nucleotide
SCI: Sister chromatid intertwine
SMC: structural maintenance of chromosomes
topo: topoisomerase
